# Role of human factors in pediatric cardiac surgery

**DOI:** 10.21542/gcsp.2016.37

**Published:** 2016-12-30

**Authors:** Akhlaque N Bhat

**Affiliations:** Department of Cardiothoracic Surgery, Section of Pediatric Cardiac Surgery, Hamad Hospital, Doha, Qatar

## Introduction

According to the Institute of Healthcare Improvement, “human factors” refers to the discipline of engineering that details the interface of people, equipment and the environment in which they work. Issues that impact human performance and increase the risk of error include factors that directly enable decision making, such as perception, attention, memory, reasoning, judgement and factors that directly enable decision execution, such as communication and the ability to carry out the intended action.

Between 2008 and 2010 around 80,000 patients had surgery for congenital heart disease in North America^1^. About 25% of these patients were neonates. The overall mortality of surgical treatment of congenital heart disease is 4%. Neonates account for 50% of the total deaths following surgical treatment, emphasising the high risk nature of this group. Neonates are especially vulnerable for a variety of reasons. During intrauterine life, fetal circulation tolerates the most severe forms of congenital heart disease. Intra and extra cardiac shunts allow fetal circulatory adaptation to abnormal heart anatomy. At birth, metabolic rate and fetal oxygen consumption increases several fold. Coexisting congenital heart disease imposes additional metabolic demands on the new born baby. Duct dependent systemic or pulmonary circulations impose hemodynamic challenges. The organ systems of the new born baby are relatively less mature. Premature birth and low birth weight can impose considerable risk. So a neonate with congenital disease who needs surgical management usually has a complex cardiac defect, is transitioning from the protective environment of the fetal circulation to a highly stressful postnatal circulation, has immature organ systems, and is facing the likely spectre of cardiopulmonary bypass with its accompanying pan-systemic effects. Congenital heart surgery in the neonatal period is a high-risk undertaking with poor error tolerance and a limited ability to retrieve^2^.

Of all the deaths in congenital heart surgery 20% are from preventable causes, implying that human factors have a significant role to play in their causation^2^. These events may or may not be preventable, but the frequency of events and the relatively focused patient population makes congenital heart surgery a model for investigating complex systems and for studying human errors and their impact on patient safety across healthcare settings^3^.

## Dynamics of poor outcomes

Preventable adverse events may occur through technical and non-technical factors associated with decision-making^4^. Technical skills are fundamental to good outcomes but non-technical skills also markedly influence the performance of individuals and teams, therefore influence the outcomes of treatment^5^. The study of human factors principally involves understanding complex systems and the relationship between people, tasks and their ever-changing environment^6^. James Reason proposed the organisational accident model to explain the dynamics of poor outcomes in complex high technology systems like the aviation industry, nuclear power industry, and healthcare industry. According to this model, a complex system like modern healthcare has a sharp end and a blunt end. The sharp end of the system is the people at the service delivery end of the system (doctors, nurses, pilots, engineers). Failures at sharp end of the system have an immediate impact on safety and are called active failures. At the blunt end of the system are higher management in the organisation e.g., administrators, regulators, governments. Failures at the blunt end of the spectrum are called latent failures. Wrong decisions made by people at the blunt end lead to weaknesses in the defences of the organisation. In this vulnerable state when active failures happen in an organisation they combine with latent failures to overcome the defences of the system and result in an organisational accident^7^. In a pediatric cardiac surgery setup examples of active failures include poor communication between surgeons, cardiologists, anesthetists and intensivists, taking patients with incorrect diagnosis to the operating room, poor scheduling of cases, technical errors during operating. Examples of latent failures include poor organisation of staff roles, absence of policies and protocols for treatment, shortage of cardiac trained nurses, lack of investment in high technology equipment and failure to invest in staff training.

## Human factors studies in pediatric cardiac surgery

Marc de Leval pioneered the study of human factors in pediatric cardiac surgery. In an important article published in the Lancet in 1987 he wrote “By and large medical world has had great difficulties in dealing with error. The traditional teaching is that medical doctors are expected to function without error. This need to perform faultlessly has created a strong pressure to intellectual dishonesty, to cover up mistakes rather than admit them, and to overlook opportunities for improvement. The reality of the malpractice threat provides strong incentives against disclosure of mistakes”^8^. As a result vital information about mistakes and opportunities to redress them are lost.

In the first ever study conducted to evaluate the role of human factors in pediatric cardiac surgery, de Leval teamed up with James Reason, the father of accident organisational theory. Using trained human factors researchers to make real-time observations during 243 arterial switch operations performed by 21 surgeons at 16 UK hospitals over an 18-month period, major and minor human failures were recorded during the course of these operations. Major failures were classified as events that were likely to have life threatening effects if left uncompensated. Minor failures were events that in isolation were not likely to have serious consequences for the patient. They concluded that minor and major events commonly occurred in pediatric cardiac surgery. The number of major events per case were strong predictors of death but proper compensation greatly reduced the risk of death. However a single uncompensated major event can lead to death.

Minor events are different because they are usually not noticed by the team members. No conscious attempt is made to compensate them. However they have a multiplicative effect, so that though in isolation they may have little impact but their multiplication has a strong relationship to negative outcomes^9^. In a subsequent analysis published by the same group they concluded that uncompensated minor events can impede a teams ability to compensate for future major events. Latent elements within the organisation predispose teams to have major events.

Paul Barach and his team studied the impact of human factors on intraoperative adverse events and compensation mechanisms in 102 patients undergoing pediatric cardiac surgery. They classified the adverse events into major and minor events. Events were further classified into *compensated* or *uncompensated* depending on whether appropriate action was taken to offset the effect of adverse event. They concluded that adverse events routinely occurred during pediatric cardiac surgery and were mostly compensated. Compensation was mostly reactionary and not preventative with possibility of prevention by improved processes.

Ninety percent of major adverse events and 90% of minor adverse events were compensated. An average of 1.2 major adverse events occurred per case, which were mostly cardiovascular events, and compensation was mostly cognitive. An average of 15.3 minor adverse events occurred per case which were mostly communication and coordination failures. The time period most vulnerable to adverse events was the period between aortic cross clamping and cross clamp release and subsequent weaning from cardiopulmonary bypass. About 45% of all adverse events occurred during this period. Case complexity and duration of surgery were predictors of major adverse events.The number of major adverse events per patient correlated with clinical outcomes^10^.

## Technical performance as a human factor

While patient outcomes are dependent on multiple factors, technical performance by a surgeon during an operation is one of the most important variables in determining outcomes in patients with congenital heart disease. Boston Children’s Hospital pioneered the development of a Technical Performance Score (TPS) to measure quality of repair after an operation. The initial study included four operations; repair of ventricular septal defect, tetralogy of Fallot, atrioventricular septal defect, and the arterial switch procedure^11^. It was later extended to assess the repair in Norwood procedure^12^.

Each of these operations was subdivided into its component sub-procedures based on the anatomic region of repair and a scoring system was developed defining three categorical variables for each sub-procedure; optimal, adequate and inadequate, based on a consensus between cardiac surgeons and cardiologists on echocardiographic assessment of each sub-procedure.

Echocardiography was used as the assessment tool because it is simple, reliable, available at the bedside and over the years has become the standard tool for assessing patients with congenital heart disease preoperatively, intraoperatively and post operatively. Optimal repair is achieved when all sub-procedures and conduction were graded as optimal and it is graded as inadequate if any component sub procedure or conduction was graded inadequate. Adequate repair figured somewhere in between the two with intact conduction as its integral component.

TPS is downgraded to match the lowest component score of the procedure. In scoring a VSD repair for example an optimal repair would include absence of a residual shunt or a shunt less than 1 mm with intact conduction, an adequate repair would include a shunt less than 3 mm with intact conduction and an in-adequate repair would include a shunt greater than 3 mm and/or a need for pacemaker or need for reintervention^13^.

Patients with inadequate TPS had significantly longer PICU and hospital stays, longer ventilation times and a higher occurrence of major postoperative complications^14^. TPS is a powerful predictor of postoperative outcome in Norwood procedure. Patients who had optimal technical scores had 1.2% hospital mortality compared to the ones with inadequate TPS whose mortality was 34.8%^11^.

In another prospective study, TPS was a strong predictor of postoperative morbidity in 166 neonates and infants who underwent a wide variety of operations for congenital heart disease with high complexity. Optimal TPS was associated with lower postoperative morbidity. Adequate TPS was associated with higher morbidity in babies with higher case complexity and lower morbidity in babies with lower case complexity. Inadequate TPS was associated with higher morbidity irrespective of case complexity^15^.

Technical performance score is emerging as an important tool to assess the quality of repair. As more and more studies emerge to show TPS as a predictor of patient outcomes in congenital heart surgery it has the potential of becoming a requirement as a quality control measurement^13^.

## Communication as a human factor

According to the Joint Commission International, 65% of sentinel events in 2009 occurred due to communication failure between various teams looking after a patient. Quite often errors result from breakdown of communication and coordination between teams^16^. Handover of a pediatric cardiac surgery patient from operation theatre to the intensive care environment is a particularly vulnerable time. This handover serves as a basis for transferring responsibility of patient care from the surgical team to the intensive care team. This vulnerability is compounded by the fact that handovers are not taught to physicians and nurses in a systematic way. Workforce constraints and limits on duty hours of physicians lead to increased handovers. Paradoxically errors in communication are more frequent in information technology-intense environments^17^.

Making handovers safe involves standardizing handovers, identifying and emphasizing key content to be handed over, monitoring handovers continuously to ensure protocol is being followed and identifying and resolving barriers to improvement of handovers^18^. Important aspects of a handover process that are essential to successful transition of patient care include limiting discussions to those related to the patient, a face-to-face sharing of patient information and discussion of plan between all care providers involved, efficient transition of equipment and limiting interruptions during the information handoff^19^.

A prospective interventional study in 79 pediatric cardiac surgery patients was undertaken to determine if implementation of standardised handover protocol from operating theatre to pediatric intensive care unit could reduce the number of errors occurring during transition of care^19^.

A single observer was responsible for data collection during all pre-intervention and post-intervention handovers using a standardized checklist. Technical errors were defined as protocol deviations involving personnel, equipment or handover process e.g. nonfunctional critical equipment at the time of patients arrival to the ICU, interruption of verbal handoff by receiving caregivers and disruption of the sterile team environment.

Information omissions were defined as failure to present protocol elements that were normally expected to be presented verbally during the handover to the receiving team e.g. procedure performed, cardiopulmonary bypass time, current inotrope infusions etc.

**Figure 1.**
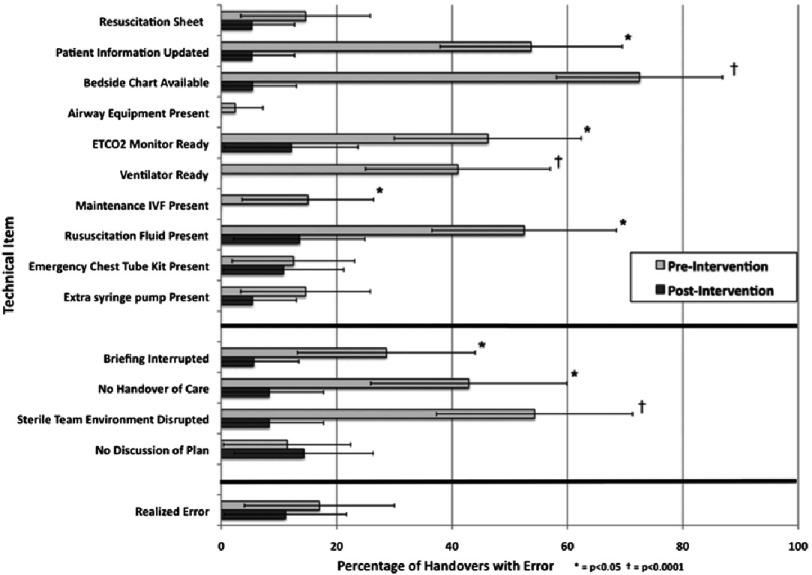
Frequency of technical errors observed before and after interventions, with 95% confidence intervals. ETCO2, end-tidal CO2; IVF, intravenous fluid. Reproduced with permission from [19].

**Figure 2.**
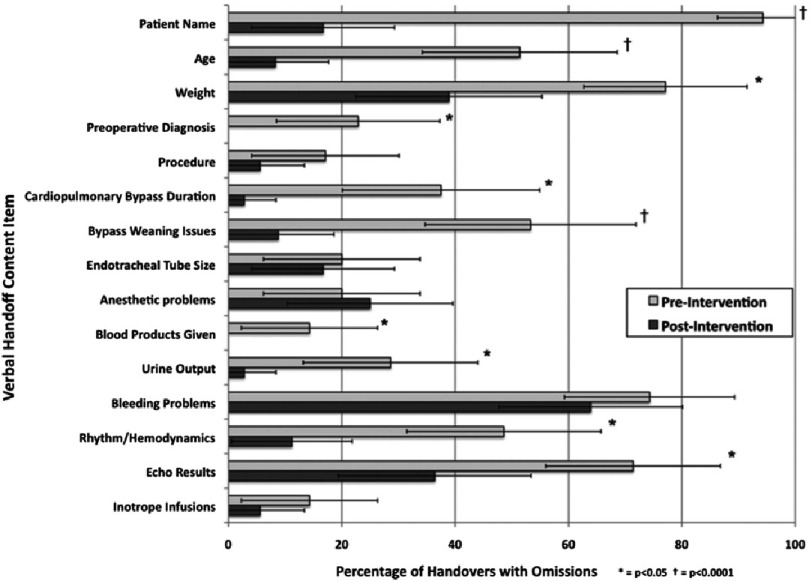
Frequency of information observed before and after intervention, with 95% confidence intervals. Reproduced with permission from [19].

Technical errors reduced from 6.24 to 1.52 and critical information omissions reduced from 6.33 to 2.38 per handover after intervention (Figures 1 & 2). There was no change in duration of handover process. The study concluded that structured handover protocol for pediatric cardiac surgery patients transitioning from operating room to the intensive care unit reduces medical errors and improves team work among care givers.

Another study used Formula 1 pit stop and aviation models to improve safety and quality of health care. This was a prospective intervention study which measured the change in performance before and after implementation of a handover protocol which was developed after exhaustive discussions between the clinicians, a Formula 1 racing team, and aviation experts. They found that implementation of the new handover protocol reduced the number of technical errors, minimised information omissions, and the duration of handovers was also reduced^20^.

## Other non-technical human factors

Management and leadership play a crucial role in creating a culture of safety. They do this by creating conditions, resources and environment in which clinicians strive to create safe outcomes. Leaders create the climate that enables clinicians to acknowledge mistakes and encourage worker-clinicians to innovate^3^.

Databases provide a platform that enables comparison of outcomes between pediatric cardiac centers which significantly contributes to quality improvement. There are four pre-requisites that ensure that multi-institutional databases provide meaningful outcomes analysis in pediatric cardiac surgery: a common nomenclature, a uniform core data set, mechanisms of auditing and validating data for accuracy and adoption of uniform risk adjustment models to evaluate case complexity^21^. The Society for Thoracic Surgeons (STS) database is one such important database in pediatric cardiac surgery that allows comparison of outcomes between centres within North America, but is unfortunately not open to centres outside the continent.

## Human factors in Qatar

Achieving excellence in healthcare in general, and in pediatric cardiac surgery in particular, requires concerted efforts by leaders and clinicians to achieve “high reliability” i.e. delivering consistent performance at high levels of safety over long periods of time. The demographics of Qatar are different from North America and Europe and the role of human factors in health care safety therefore presents unique challenges for the following reasons:

1. A vast majority of health care personnel including physicians, nurses, technicians and healthcare administrators belong to diverse nationalities and backgrounds. Their training therefore vastly differs. Their skill and experience is molded by the society and environment they grew up in. Many healthcare personnel are unlikely to have received formal instruction in perception, assessment and mitigation of risk and error. Their perception of risk and error in context of the patient care is therefore likely to differ as well.
2. Verbal communication between healthcare providers poses a particularly important challenge in providing safe healthcare to patients. In Qatar, English is the unifying language for healthcare workers who speak more than seventy different mother tongues. English language is therefore spoken with many different accents and colloquialisms. This creates a risk wherein vital information can get miscomprehended or lost in translation at routine handovers or in acute situations such as coordinating management of patients in life threatening situations. Reluctance to ask a colleague to repeat him- or herself for fear of offending them can leave large gaps in information. This problem becomes even more difficult when information is exchanged on the telephone.

Dealing with these unique challenges is vital to the development of safe healthcare in Qatar. There is a strong need to develop a culture of safety within this diverse group of healthcare providers fostered by a supportive leadership. One of the most important challenges is how to develop a culture that supports the changes that are required to enable learning.

In that regard, establishment of Hamad Healthcare Quality Institute by the Hamad Medical Corporation is a big step in the right direction. Establishment of Hamad Healthcare Quality Institute will enable our organisation to understand our current healthcare safety culture and review how teams can work together to improve the quality of health care we provide . It will design and develop evidence based practical solutions for patients and promote access to high quality safe healthcare services across Qatar^22^.

## Conclusion

Pediatric cardiac surgery has become a model for the delivery of complex multidisciplinary care. For optimal outcomes pediatric cardiac surgery relies on an organisational structure that allows early identification of deficiencies through extreme vigilance and eliminates deficiencies through use of process improvement. In other words the specialty of pediatric cardiac surgery in its ideal form exists within a “culture of safety”. To quote Marc de Leval “safety is not a commodity, but a value that requires continuous reinforcement and investment”^23^.
